# Attentional Blink in Pilots and Its Relationship With Flight Performance

**DOI:** 10.3389/fpsyg.2020.01696

**Published:** 2020-07-14

**Authors:** Fengzhan Li, Quanhui Liu, Huijie Lu, Xia Zhu

**Affiliations:** Department of Military Medical Psychology, Air Force Medical University, Xi’an, China

**Keywords:** military pilot, attentional blink, flight performance, cognitive ability, attention

## Abstract

**Objective:**

In flight, military pilots need to monitor changes in the external environment and monitor the situation of the aircraft at the same time. Attentional blink (AB) reflects attentional blindness in time. Therefore, the present study investigated the AB effect in military pilots and its relationship with flight performance.

**Methods:**

Thirty male military pilots (44.23 ± 4.07 years old) and 29 control participants (44.07 ± 2.93 years old) underwent testing with the classic rapid serial visual presentation paradigm. The participants’ accuracy in detecting a second target stimulus (T2/T1) on the basis of their correctly response to the first target stimulus (T1) was calculated to measure the AB effect. The flight performance of these military pilots was also collected.

**Results:**

The participants’ accuracy in detecting T2/T1 at positions of 180, 270, 360, and 450 ms was significantly lower than that in detecting T1 in both groups. The military pilots’ detection accuracy of T2/T1 at the positions of 180 ms (*p* < 0.001) and 270 ms (*p* < 0.001) was significantly higher than that of the control participants, and their mean detection accuracy of T2/T1 (AB effect) at the positions of 180, 270, 360, and 450 ms was also significantly higher than that of the control participants (*p* < 0.001). There was a significant correlation between the AB effect and the lowest flight performance score for the military pilots (*r* = 0.52, *p* = 0.004), and the regression coefficient was significant (β = 0.514, *p* = 0.004, *R*^2^ = 0.31).

**Conclusions:**

Both groups experienced the AB effect, but the military pilots’ performance regarding the AB effect was better than that of the control participants. The AB effect can predict the lowest flight performance score in military pilots. These findings may have implications for the grounding and selection of Chinese military pilots.

## Introduction

When multiple stimuli appear sequentially in the same location, if an individual detects the first target stimulus correctly, his or her accuracy in detecting a second target stimulus that appears within approximately 200–500 ms after the first target stimulus is low. This phenomenon is called attentional blink (AB; Raymond et al., 1992). It reflects attentional blindness in time. There are many theoretical explanations for AB, such as inhibition theories (Dux and Marois, 2008), the temporary loss of control hypothesis (Di Lollo et al., 2005), and a two-stage model (Chun and Potter, 1995). In summary, there are two main theories (Dux and Marois, 2009). One of the main theories is that the emergence of AB is related to cognitive resources being limited. Individuals consume many cognitive resources when they detect the first target stimulus, so it very difficult for them to fully process the second target stimulus or allow the second target stimulus to be encoded into their working memory. The other main theory is that the AB effect is related to an individual’s ability to suppress distraction stimuli. The number of distraction stimuli varies across target stimuli. It may be difficult for individuals to exclude them by cognitive control, resulting in a decline in their accuracy in detecting a second target stimulus.

The AB effect differs across various populations. Many studies have shown that the AB effect in social anxiety patients (Morrison et al., 2016), alcoholics (DePalma et al., 2017), autism patients (Amirault et al., 2009), and patients with schizophrenia (Mathis et al., 2011) are more severe. These populations generally have attention problems or executive cognitive defects, which prevent them from detecting target stimuli effectively. However, the AB performance of some athletes and players, such as shooting athletes (Mao, 2016), table tennis athletes and soccer players (Li, 2002; Li and Zhang, 2004), martial arts athletes (Liu, 2008), Sanda athletes (Wang and Zhou, 2010), and video game players (Green and Bavelier, 2003; Li et al., 2015), is better than that of ordinary people. This phenomenon might be attributed to their long-term experiences in training and playing games. Because of the intense, competitive environment, individuals must identify and judge rapidly changing information correctly by being attentive; otherwise, they lose the game.

As with high-performance athletes and video game players, pilots have to perform a difficult task that demands a large amount of attentional resources, i.e., flying a plane. In flight, military pilots need to monitor changes in the external environment and monitor the situation of the aircraft at the same time. Military pilots need to identify and filter many kinds of complex information immediately and correctly in a short period (Johnson et al., 2017). They must act appropriately, especially when they have to handle a variety of emergencies. Otherwise, accidents can easily occur in flight. Then, are military pilots’ attentional abilities as high as those of high-performance athletes and video game players? Is military pilots’ AB performance better than that of ordinary people? Additionally, detecting information correctly in a short period is a basic prerequisite for military pilots to avoid accidents in flight (O’Hare, 2006). Therefore, can the AB effect predict flight performance? There are few related studies about these questions. The aim of the present study was to investigate them.

## Materials and Methods

### Participants

The present study mainly focused on males because the majority of military pilots are male. Thirty male military pilots and 29 male control participants participated in the study. The pilots were 44.23 ± 4.07 years old. Their flight times were 3402.10 ± 1196.12 h, and the types of aircrafts that they flew were fighters (63.3%), trainers (20%), bombers (10%), and airfreighters (6.7%). The control participants were 44.07 ± 2.93 years old and were administrative staff members of the Air Force. Each participant’s visual acuity or corrected visual acuity was normal, without color vision deficiencies or color blindness. All participants were in good health, with no histories of major physical diseases or mental disorders.

### Design and Procedure

The classic rapid serial visual presentation (RSVP) paradigm was adopted in the present study (Martens and Wyble, 2010); 10 stimuli, including several numbers and two letters, were displayed each second on a computer screen. The numbers were the distraction stimuli, while the letters were the target stimuli that the subjects were required to detect correctly. It is worth mentioning that to eliminate interference between numbers and letters, 0, 1, I, O, Q and Z were not included in the experiment. The background color of the computer screen was black. The colors of all the stimuli were white, the text font was Courier New, and the text size was 40.

**FIGURE 1:**
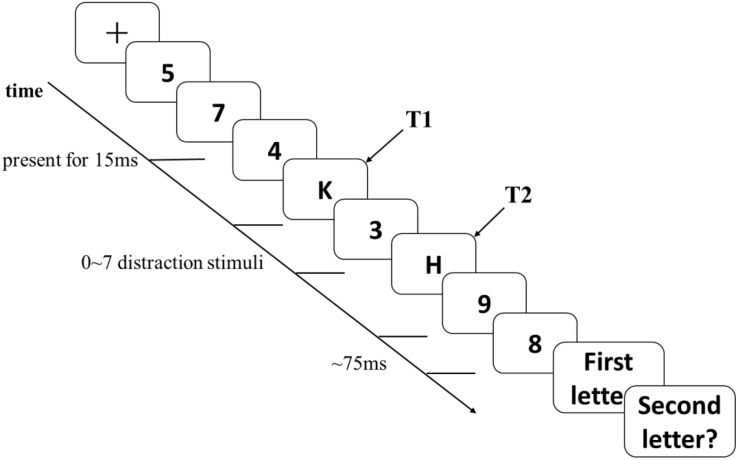
The classic rapid serial visual presentation paradigm.

The RSVP paradigm included two blocks. The first block, i.e., the practice session, contained eight trials; the second block, i.e., the formal experiment, comprised 72 trials. The experimental design for each trial is shown in Figure 1. First, a fixation point (“+”) was presented on the computer screen for 1000 ms. Then, a series of numbers and letters were continuously presented at the position of the fixation point. Two letters, which were the target stimuli, were displayed in each trial. Each number or letter shown on the computer screen was presented for 15 ms. The interval between stimuli was 75 ms. There were four to six numbers (the distraction stimuli) that were displayed before the first letter appeared (the first target stimulus, denoted as T1). Then, four to six numbers were displayed after the second letter appeared (the second target stimulus, denoted as T2). Between T1 and T2, there were various numbers of distraction stimuli, ranging from zero to seven. In other words, T2 appeared in the first to the eighth position after T1. All the letters and numbers appeared randomly. The entire experimental procedure was implemented in E-Prime Studio 3.0 on 14-inch, 1920 × 1080 HD LED ThinkPad monitors at a 60 Hz refresh rate.

Primarily, the participants were asked to sign an informed consent form and fill out a basic information questionnaire. The pilot’s basic information included sex, age, flight time, and flight performance. The pilots’ flight performance, such as his accuracy in performing special flight maneuvers and flight course, was assessed by the supervisor twice a year according to the Flight Assessment Outline established by the Chinese Air Force. The pilots received one comprehensive score based on the internal scoring system after each flight assessment. In the present study, flight performance included the highest, average, and lowest scores over the past 5 years. The control participants’ basic information included sex and age. Then, both groups underwent testing with the RSVP paradigm. During the experimental session, the participants were approximately 50 cm away from the computer screen, which was a comfortable distance for them.

The participants needed to immediately identify the two target stimuli after each trial was presented and type them into the computer in order. The present study mainly investigated participants’ accuracy in detecting T2 on the basis of a correct response to T1 (T2/T1). If T2 appeared immediately after T1, there was no distraction stimulus between T1 and T2, and the accuracy in detecting T2/T1 was recorded as lag1 (90 ms). If there was a distraction stimulus between T1 and T2, then the accuracy of T2/T1 was recorded as lag2 (180 ms). If there were two distraction stimuli between T1 and T2, then the accuracy of T2/T1 was recorded as lag3 (270 ms). The remaining data were recorded in the same manner until lag8 (720 ms), which means that there were 7 distraction stimuli between T1 and T2.

### Statistical Analysis

Data processing and statistical analyses were conducted in SPSS 22.0. ANOVA, correlation analysis and regression analysis were conducted in the present study.

## Results

### Attentional Blink Effects Were Observed in Both Groups

**FIGURE 2:**
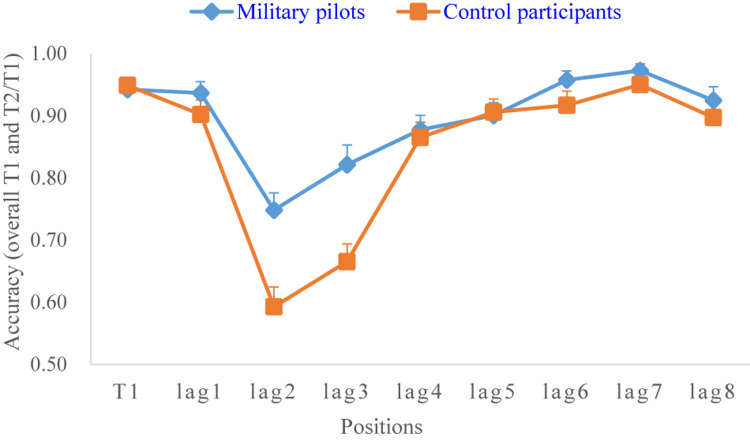
A line chart showing the accuracy for both groups.

To examine whether the military pilots experienced an AB effect, their accuracy in detecting the target stimuli (the overall accuracy for T1 and the accuracy for T2/T1 at each position) was analyzed by one-way repeated measures ANOVA (positions: T1 vs lag1 vs lag2 vs lag3 vs lag4 vs lag5 vs lag6 vs lag7 vs lag8). The results showed that the main effect of position was significant (*F* = 8.84, *p* < 0.001, *m*η^2^ = 0.76). The results of the *post hoc* test, where the overall accuracy of T1 was used as the baseline level for comparison, showed that the accuracy values for only lag2 (180 ms), lag3 (270 ms), lag4 (360 ms), and lag5 (450 ms) were significantly lower than the overall accuracy for T1 (Table 1). Similarly, one-way repeated measures ANOVA with position as a within-subject factor was also conducted on the control groups. The results showed that the main effect of position was significant (*F* = 30.57, *p* < 0.001, mη^2^ = 0.92), and the results of the *post hoc* test also showed that the accuracy values for only lag2 (180 ms), lag3 (270 ms), lag4 (360 ms), and lag5 (450 ms) were significantly lower than the overall accuracy for T1 (Table 1). Both lines on the chart had a “U” shape (Figure 2). These results indicated that both groups experienced an AB effect during 180–450 ms.

**TABLE 1:** Results of the *post hoc* tests in which the overall accuracy for T1 was used as the baseline level for comparison.

T1 - T2/T1	Military pilots		Control participants	
		
	*M*_T1–T2/T1_	SD	*M*_T1–T2/T1_	SD
T1-lag1	0.01	0.02	0.05	0.02
T1-lag2	0.19**	0.03	0.36**	0.03
T1-lag3	0.12**	0.03	0.28**	0.03
T1-lag4	0.06**	0.02	0.08**	0.02
T1-lag5	0.04*	0.02	0.04*	0.02
T1-lag6	–0.02	0.01	0.03	0.02
T1-lag7	–0.03	0.01	0.00	0.01
T1-lag8	0.02	0.02	0.04	0.03

*p < 0.05, **p < 0.01. T1 was the participants’ overall accuracy in detecting the first target stimulus, and T2/T1 was their accuracy in detecting the second target stimulus. lag1 was the accuracy for T2/T1 at the position of 90 ms, and lag2 was the accuracy for T2/T1 at the position of 180 ms. lag4∼lag8 were recorded in the same manner.

Therefore, the present study considered the mean accuracy values for lag2, lag3, lag4, and lag5 as measures of the AB effect. The formula used to calculate this measure was AB = (lag2 + lag3 + lag4 + lag5)/4. The larger the AB value was, the better the performance regarding the AB effect was.

### The AB Performance of the Military Pilots Was Better Than That of the Control Participants

To compare the AB performance between the two groups, the overall accuracy of T1 and T2/T1 at each position and the AB value are shown in Table 2. Li (2002) proposed that if the accuracy for T2/T1 was greater than 95%, there was no AB effect. If it was between 85 and 95%, there was a slight AB effect. If it was 75–85%, there was a mild AB effect. If it was 65–75%, there was a moderate AB effect. If it was 55–65%, there was a severe AB effect. If it was lower than 55%, there was a complete AB effect. According to this classification, the results in Table 2 show that the military pilots experienced stages of slight and mild AB successively, and the control participants experienced stages of severe, moderate and mild AB successively.

**TABLE 2:** The analysis of the simple effects.

	*M*_pilots_	*M*_control_	SD	SD
T1	0.94	0.95	–0.01	0.01
lag1	0.94	0.90	0.04	0.03
lag2	0.75	0.59	0.16***	0.04
lag3	0.82	0.67	0.15***	0.04
lag4	0.88	0.87	0.01	0.03
lag5	0.90	0.91	–0.01	0.03
lag6	0.96	0.92	0.04	0.03
lag7	0.97	0.95	0.02	0.02
lag8	0.92	0.91	0.01	0.03
AB	0.84	0.76	0.08***	0.02

***p < 0.001.

More specifically, the overall accuracy for T1 and T2/T1 at each position and the AB value were examined with 2 × 10 (groups: pilots vs control) (T1 vs lag1 vs lag2 vs lag3 vs lag4 vs lag5 vs lag6 vs lag7 vs lag8 vs AB) mixed-design ANOVA, with group as a between-subjects factor. The results showed that the main effect of the between-subjects factor was significant (*F* = 36.52, *p* < 0.001, *m*η^2^ = 0.85), and the interaction effect was also significant (*F* = 4.77, *p* < 0.001, *m*η^2^ = 0.43). Then, the analysis of the simple effect showed that there was no significant difference between the two groups in the overall accuracy for T1 (Table 2), indicating that they had the same baseline level. However, the lag2 and lag3 values for the military pilots were significantly higher than those for control participants, and the pilots’ AB performance was also significantly higher than that of the control participants (Table 2). These results indicated that the AB performance of military pilots is better than that of the control participants.

### The Relationship Between the AB Effect and Flight Performance

**FIGURE 3:**
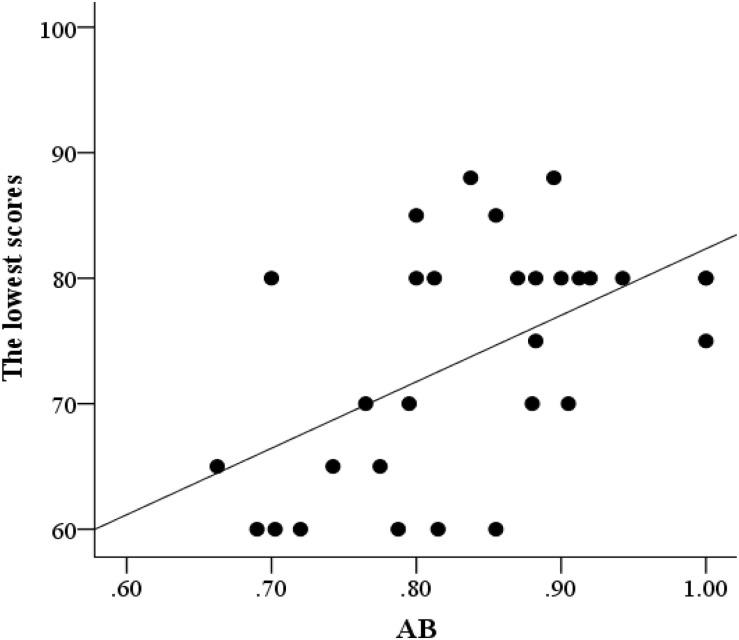
A scatter plot of the relationship between AB and the lowest flight performance scores.

**FIGURE 4:**
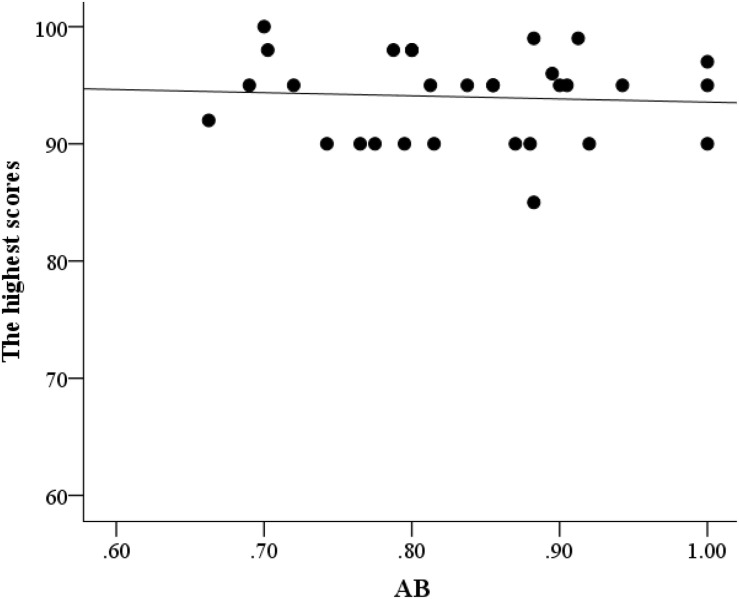
A scatter plot of the relationship between AB and the highest flight performance scores.

**FIGURE 5:**
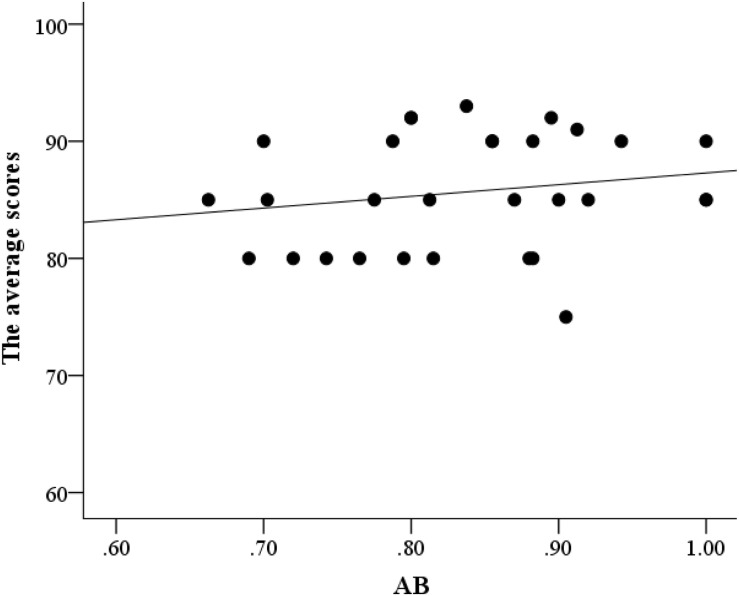
A scatter plot of the relationship between AB and the average flight performance scores.

Partial correlation analysis was conducted between AB and flight performance, with age and flight time serving as control variables. The AB effect was significantly correlated with the lowest score (*r* = 0.52, *p* = 0.004; Figure 3) but not with the highest (*r* = –0.09, *p* = 0.65; Figure 4) and average scores (*r* = 0.18, *p* = 0.36; Figure 5). This finding indicated that better AB performance was associated with the lowest flight performance score.

Then, a regression analysis was conducted, with the AB effect as the predictive variable and the lowest score as the dependent variable. When age and flight time were considered control variables, the regression effect was significant, with a standardized regressive coefficient of β = 0.514 (*p* = 0.004 and *R*^2^ = 0.31) (Table 3). This finding indicated that the AB effect of military pilots can significantly predict the lowest flight performance score.

**TABLE 3:** Regression analysis of AB and the lowest flight performance score.

	β	*t*	*p*	95% CI of B	
				
				Lower bound	Upper bound
Constant		2.004	0.056	–1.210	95.931
Age	–0.167	–0.813	0.424	–1.345	0.583
Flight time	0.005	0.025	0.981	–0.003	0.003
AB	0.514**	3.132	0.004	17.693	85.247

***p < 0.001, β is the standardized regressive coefficient, CI is the confidence interval.

## Discussion

Attention is the basis of human information processing. The AB effect suggests that when people correctly identify some information, their ability to accurately identify subsequent stimuli is affected. Raymond et al. (1992) found that if subjects detect the first target stimulus correctly, the distribution diagram of their accuracy in detecting a second target stimulus forms a “U” shape. In other words, the correct detection of the first stimulus has a negative influence on individuals’ accuracy in detecting the second target stimulus. This finding has been reported in a large number of studies (Popple and Levi, 2007; MacLean and Arnell, 2012; Dale et al., 2013), and it seems to be a phenomenon that is difficult to avoid. The present study supports these findings as well. In the present study, both groups experienced the AB effect.

However, the military pilot’s AB performance was better than that of the control group, and the AB effect could predict the lowest flight performance score among the military pilots. Theoretically, the AB effect may reflect an individual’s working memory and his or her ability to suppress distraction stimuli, both of which are essential for military pilots (Martins, 2016). As complex advancements in the airplane cockpit have been made to provide more information and make it more intelligent, the information provided by the display and control system has also become diverse and complex. A variety of information is available to provide military pilots with comprehensive and accurate data about airplane cockpit conditions and the external environment (Cheng et al., 2019). However, these advancements lead to a new problem; some key information may be hidden or overlooked accidently among the large amount of display information. It is therefore difficult for military pilots to select and perceive the information accurately (O’Hare, 2006), which can result in some human errors and catastrophic consequences in practice. Therefore, military pilots need to monitor internal and external environmental information in real time with their working memory and inhibition abilities. They need to identify and judge the conditions of instruments immediately (Wu et al., 2015). They must collect and integrate all types of information to make appropriate decisions while dismissing distraction stimuli (Yu et al., 2016). In short, good AB performance among military pilots ensures that they are able to obtain as much information as possible and reduces the probability that they will miss important information. Without a doubt, this phenomenon may be related to their long-term flight training and experience.

The result in this study showing that AB can predict flight performance is consistent with previous findings, which indicates that AB can predict the driving performance (Trick et al., 2008) and flight performance (Du et al., 2015). Nevertheless, it is interesting to note that the present study showed that the AB effect has a significant correlation with the lowest score but not the highest and average scores of flight performance. In other words, the lowest flight performance scores can be predicted by the military pilot’s AB effect. This finding indicates that the AB effect is not limited to being a measure of military pilots’ competency, but it may be one of the objective indicators for predicting possible accidents in flight. Xing and Bailey (2005) found that 60% of flight operation errors were related to attention and memory, and the AB effect was one of the three major reasons. The more serious the AB effect is, the more likely that an accident will occur. After all, when pilots do not miss any information during flight, especially key information, flight safety is guaranteed.

Therefore, the results of the present study suggest there is an objective indicator to determine whether military pilots need to be grounded. At present, there are three major reasons for Chinese military pilots need to be grounded. The first reason is physical illness, such as problems with the eyes and spine (Wang and Zhan, 2019). The second reason is related to psychological factors, such as mental health (Qi et al., 2019), emotion (Lin and Zhang, 2001), personality (Yi and Huang, 2011), and spatial disorientation (Zhang, 2011). The last reason is related to age. For example, according to the latest version of Provisions on Administration of Flight Personnel, military pilots who are in a certain age range are considered for grounding. However, it may be controversial to ground for some pilots who are at the lower limit of the age range. In addition, more information other than age may be needed. For example, individuals’ cognitive performance needs to be examined after physical and psychological problems are excluded. For instance, some pilots may be younger than a certain age, but their AB performance has declined. If they continue to fly a plane, the number of flight errors that occur might increase. Conversely, other pilots may be older than a certain age, but they are in good condition, and their AB performance is still good, indicating that they are able to continue flying. If they are grounded, it may be a waste of manpower. Therefore, the AB effect can be used as an indicator for military pilots to be grounded to prevent the occurrence of flight accidents and improve the selection of pilots for grounding.

In addition, it may also be beneficial for the selection of military pilots to use the AB effect as one criterion. The cost of training a military pilot is very high (Li, 1983; Fu, 2000). It takes at least 6–8 years to become a qualified pilot from a flying cadet. Therefore, it may be that some cadets who have poor AB performance should not be recruited. It will take more time, effort, and financial resources to train them to fly planes safely. If we put effort and financial resources into training the potential cadets and exclude those who cannot suppress distraction stimuli or are likely to miss key information, training efficiency will greatly improve. However, these assumptions need to be further verified by longitudinal studies in the future.

## Conclusion

In conclusion, the present study showed that both military pilots and ordinary people experience the AB effect. However, the military pilot’s AB performance was better than that of the control participants, and the AB effect can predict the lowest flight performance among military pilots. These findings may have implications for the grounding and selection of Chinese military pilots.

## Data Availability Statement

All datasets presented in this study are included in the article/supplementary material.

## Ethics Statement

The studies involving human participants were reviewed and approved by First Affiliated Hospital of Air Force Medical University. The patients/participants provided their written informed consent to participate in this study.

## Author Contributions

FL conceived the study and wrote the manuscript. XZ, QL, and HL critically reviewed the drafts of the manuscript. All authors contributed to the article and approved the submitted version.

## Conflict of Interest

The authors declare that the research was conducted in the absence of any commercial or financial relationships that could be construed as a potential conflict of interest.

## References

[B1] Amirault M. Delord S. Mendizabal S. Kraushaar C. Hesling I. Allard M. (2009). Alteration of attentional blink in high functioning autism: a pilot study. *J. Autism. Dev. Disord.* 3 1522–1528. 10.1007/s10803-009-0821-5 19636692

[B2] Cheng N. Wu H. Qiu Z. (2019). Modeling interaction between pilot and airborne computer and research on its problems. *Aeronaut. Comput. Tech.* 49 105–108.

[B3] Chun M. M. Potter M. C. (1995). A two-stage model for multiple target detection in rapid serial visual presentation. *J. Exp. Psychol. Hum. Percept. Perform.* 21 109–127. 10.1037/0096-1523.21.1.109 7707027

[B4] Dale G. Dux P. E. Arnell K. M. (2013). Individual differences within and across attentional blink tasks revisited. *Attent. Percept. Psychophys.* 75 456–467. 10.3758/s13414-012-0415-8 23319149

[B5] DePalma F. M. Ceballos N. Graham R. (2017). Attentional blink to alcohol cues in binge drinkers versus non-binge drinkers. *Addict. Behav.* 73 67–73. 10.1016/j.addbeh.2017.04.020 28494384

[B6] Di Lollo V. Kawahara J. Ghorashi S. M. S. Enns J. T. (2005). The attentional blink: resource depletion or temporary loss of control? *Psychol. Res.* 69 191–200. 10.1007/s00426-004-0173-x 15597184

[B7] Du F. Zhang J. Dai M. (2015). “Attentional switch characteristics are correlated with the performance of simulated aviation task,” in *Proceedings fo International Conference on Human-Computer Interaction*, (Cham: Springer).

[B8] Dux P. E. Marois R. (2008). Distractor inhibition predicts individual differences in the Attentional Blink. *PLoS One* 3:e3330. 10.1371/journal.pone.0003330 18833325 PMC2553194

[B9] Dux P. E. Marois R. (2009). The attentional blink: a review of data and theory. *Attent. Percept. Psychophys.* 71 1683–1700. 10.3758/app.71.8.1683 19933555 PMC2915904

[B10] Fu S. (2000). Measuring system for pilots’ psychological selection. *China Sci. Technol. Resour. Rev.* 7 23–26.

[B11] Green C. S. Bavelier D. (2003). Action video game modifies visual selective attention. *Nature* 423 534–537. 10.1038/nature01647 12774121

[B12] Johnson J. F. Barron L. G. Carretta T. R. Rose M. R. (2017). Predictive validity of spatial ability and perceptual speed tests for aviator training. *Int. J. Aerospace Psychol.* 27 109–120. 10.1080/24721840.2018.1442222

[B13] Li L. (1983). The selection of military pilots. *Psychol. Sci.* 6 299–304.

[B14] Li R. W. Ngo C. V. Levi D. M. (2015). Relieving the attentional blink in the amblyopic brain with video games. *Sci. Rep.* 5:8483. 10.1038/srep08483 PMC4341194 25715870

[B15] Li Y. (2002). Preliminary research on characteristics of attentional blink in high-level athletes of different attention type. *J. Beijing Univ. Phys. Educ.* 25 43–46.

[B16] Li Y. Zhang H. (2004). Experimental Study on the characteristics of female soccer players’ attentional blink. *J. Beijing Sport Univ.* 27 1193–1195.

[B17] Lin Y. Zhang D. (2001). The important emotional disorder leading to grounding-excessive tension and anxiety. *J. China Civil Aviat. Flying Coll.* 12 24–26.

[B18] Liu J. (2008). Study on the characteristics of attention blink of female taekwondo athletes. *China Sport Sci. Technol.* 42 60–64.

[B19] MacLean M. H. Arnell K. M. (2012). A conceptual and methodological framework for measuring and modulating the attentional blink. *Attent. Percept. Psychophys.* 74 1080–1097. 10.3758/s13414-012-0338-4 22821263

[B20] Mao H. (2016). *Research on the Influence of Attention Fatigue and Anxiety to Attentional Blink in Shooting Athletes and Its’ Applying.* Unpublished Master Thesis, Henan University, Kaifeng.

[B21] Martens S. Wyble B. (2010). The attentional blink: past, present, and future of a blind spot in perceptual awareness. *Neurosci. Biobehav. Rev.* 34 947–957. 10.1016/j.neubiorev.2009.12.005 20025902 PMC2848898

[B22] Martins A. P. G. (2016). A review of important cognitive concepts in aviation. *Aviation* 20 65–84. 10.3846/16487788.2016.1196559

[B23] Mathis K. I. Wynn J. K. Breitmeyer B. Nuechterlein K. H. Green M. F. (2011). The attentional blink in schizophrenia: isolating the perception/attention interface. *J. Psychiatr. Res.* 45 1346–1351. 10.1016/j.jpsychires.2011.04.002 21550051 PMC3183173

[B24] Morrison A. S. Brozovich F. A. Lakhan-Pal S. Jazaieri H. Goldin P. R. Heimberg R. G. (2016). Attentional blink impairment in social anxiety disorder: depression comorbidity matters. *J. Behav. Ther. Exp. Psychiatry* 50 209–214. 10.1016/j.jbtep.2015.08.006 26370394 PMC4679612

[B25] O’Hare D. (2006). Cognitive functions and performance shaping factors in aviation accidents and incidents. *Int. J. Aviat. Psychol.* 16 145–156. 10.1207/s15327108ijap1602_2

[B26] Popple A. V. Levi D. M. (2007). Attentional blinks as errors in temporal binding. *Vis. Res.* 47 2973–2981. 10.1016/j.visres.2007.06.022 17888482 PMC2072812

[B27] Qi J. Yu D. Liu J. Wang J. (2019). Aviation medical identification and clinical analysis of depression disorders in grounded military aircrew from 2004 to 2016. *Med. J. Air Force* 35 467–469.

[B28] Raymond J. E. Shapiro K. L. Arnell K. M. (1992). Temporary suppression of visual processing in an RSVP task: an attentional blink? *J. Exp. Psychol.* 18 849–860. 10.1037/0096-1523.18.3.849 1500880

[B29] Trick L. Brandigampola S. Enns J. (2008). Does the prolonged attentional blink to emotional stimuli affect driving performance? *J. Vis.* 8:15 10.1167/8.6.15

[B30] Wang X. Zhan S. (2019). Meta-analysis of disease spectrum associated with flying disqualification of Chinese military pilots. *Med. J. Air Force* 35 293–296.

[B31] Wang X. Zhou C. (2010). Research on attentional blink character and neural mechanism of female sanda athlete. *China Sport Sci. Technol.* 46 76–78.

[B32] Wu Q. Zhang X. Qin Z. Wang Z. Ma Y. (2015). Human agility: a new requirement to modern fighter aircraft pilot. *Chin. J. Aerospace Med* 26 161–166.

[B33] Xing J. Bailey L. L. (2005). Attention and memory in air traffic control tasks. *J. Vis.* 5 427 10.1167/5.8.427

[B34] Yi S. Huang D. (2011). Analysis of the test results of personality characteristics in flying college students. *J. Aerospace Med.* 22 947–948.

[B35] Yu C. Wang E. M. Li W. Braithwaite G. Greaves M. (2016). Pilots’ visual scan patterns and attention distribution during the pursuit of a dynamic target. *Aerospace Med. Hum. Perform.* 87 40–47. 10.3357/amhp.4209.2016 26735232

[B36] Zhang T. (2011). *Research on the Evaluation of Military Pilots’ Brain Image Under Stress.* Unpublished Master Thesis, Fourth Military Medical University, Xi’an.

